# SpliceTools, a suite of downstream RNA splicing analysis tools to investigate mechanisms and impact of alternative splicing

**DOI:** 10.1093/nar/gkad111

**Published:** 2023-03-02

**Authors:** Erik K Flemington, Samuel A Flemington, Tina M O’Grady, Melody Baddoo, Trang Nguyen, Yan Dong, Nathan A Ungerleider

**Affiliations:** Department of Pathology, Tulane University, New Orleans, LA, USA; Tulane Cancer Center, Tulane University, New Orleans, LA, USA; Department of Pathology, Tulane University, New Orleans, LA, USA; Department of Pathology, Tulane University, New Orleans, LA, USA; Department of Pathology, Tulane University, New Orleans, LA, USA; Department of Structural and Cellular Biology, Tulane University, New Orleans, LA, USA; Department of Pathology, Tulane University, New Orleans, LA, USA

## Abstract

As a fundamental aspect of normal cell signaling and disease states, there is great interest in determining alternative splicing (AS) changes in physiologic, pathologic, and pharmacologic settings. High throughput RNA sequencing and specialized software to detect AS has greatly enhanced our ability to determine transcriptome-wide splicing changes. Despite the richness of this data, deriving meaning from sometimes thousands of AS events is a substantial bottleneck for most investigators. We present SpliceTools, a suite of data processing modules that arms investigators with the ability to quickly produce summary statistics, mechanistic insights, and functional significance of AS changes through command line or through an online user interface. Utilizing RNA-seq datasets for 186 RNA binding protein knockdowns, nonsense mediated RNA decay inhibition, and pharmacologic splicing inhibition, we illustrate the utility of SpliceTools to distinguish splicing disruption from regulated transcript isoform changes, we show the broad transcriptome footprint of the pharmacologic splicing inhibitor, indisulam, we illustrate the utility in uncovering mechanistic underpinnings of splicing inhibition, we identify predicted neo-epitopes in pharmacologic splicing inhibition, and we show the impact of splicing alterations induced by indisulam on cell cycle progression. Together, SpliceTools puts rapid and easy downstream analysis at the fingertips of any investigator studying AS.

## INTRODUCTION

Alternative splicing (AS) is a fundamental regulatory feature of eukaryotic cells that plays a role in cell signaling and cell fate decisions (e.g. (1)). Disrupted splicing due to mutations at genomic splice sites or mutation of splicing factors are at the root of both germline and somatic diseases ranging from cystic fibrosis and Duchenne muscular dystrophy to cancer (2–7). Understanding the mechanisms regulating splicing in both normal physiology and disease is of great importance for understanding normal cell signaling, for elucidating the mechanistic underpinnings of splicing deficiencies in disease, and for the development of therapeutics targeting splicing defects. Further, there is substantial interest in pharmacologic splicing inhibition as a synthetic lethal approach in cancer therapeutics and for prompting the generation of neo-antigens for immune-based cancer therapies (8,9).

Numerous tools have been developed to deconvolve statistically significant splicing differences between conditions from RNA-seq data (10–14) and our ability to reliably identify splicing changes is advancing as RNA-sequencing depth increases and software development continues. Despite improvements in the accurate detection of splicing changes, little attention has been devoted to the development of downstream analysis software to decipher the complexion, the underlying mechanisms, and the functional significance of AS. This leaves many investigators with lists of hundreds to thousands of AS events with little knowledge of how to extract the significance of these alterations. To interpret their data, investigators frequently need to recruit a bioinformaticist to perform custom data analyses. Yet this is still limiting without some knowledge of what can be derived from these experiments and at least basic awareness of bioinformatic approaches to extract meaningful information. To overcome this bottleneck, we present SpliceTools, an easy-to-use software suite that enables investigators to harvest general AS characteristics, investigate the mechanistic basis of splicing changes, and explore the functional consequences of AS in their model systems. SpliceTools is available through an online web interface (splicetools.org) or for download at (https://github.com/flemingtonlab/SpliceTools).

## MATERIALS AND METHODS

### RNA-seq data acquisition and processing

RNA-seq data from 186 RBP knockdowns (15), UPF1, SMG6 and SMG7 knockdown, and indisulam and ms023 treatments (8) were downloaded from NCBI Sequence Read Archive or ENCODE (See Supplementary File S1 for accession numbers). Reads were aligned to the human hg38 assembly using STAR v2.5.2a (16) using the following settings: –chimSegmentMin 2 –outFilterMismatchNmax 3 –alignEndsType EndToEnd –outSAMstrandField intronMotif –alignSJDBoverhangMin 6 –alignIntronMax 300000. A3SS, A5SS, MXE, RI, and SE splicing changes were analyzed using rMATS 4.1.1 (10–12) with option, –libType fr-firststrand.

Gene expression [transcripts per million (TPM)] values were determined by using Kallisto v0.46.0 (17) with the –fr-stranded setting, aligning reads to the human (Ensembl 90) transcriptome.

### SpliceTools analyses

An FDR threshold of 0.0005 and rMATS JCEC (Junction Counts and Exon Coverage) files were used for all analyses. For SEIntronExonSizes, SENumberSkipped, SETranslateNMD, SEUnannotated, and RIIntronExonSizes, the human Ensembl 90 annotation in bed format with gene ID listed as Ensembl transcript ID and HUGO gene name separated by an underscore (available at https://github.com/flemingtonlab/SpliceTools) was used. For SESpliceSiteScoring, SETranslateNMD, and RISpliceSiteScoring, an hg38 human genome fasta file containing all chromosomes was used. For SEFractionExpressed and RIFractionExpressed, expression files with gene IDs in the first column followed by separate columns with TPM values (generated using Kallisto) for control conditions and then test conditions was used.

### Alternative splicing input files

SpliceTools was designed to use rMATS input JCEC files as input. The relevant information extracted from rMATS A3SS, A5SS, RI, and SE files are HUGO gene name (column 3), chromosome (column 4), strand (+/-, column 5), 0 based coordinates in columns 6–11, FDR (column 20) and Inclusion level difference (column 23) (see example files HERE for coordinate order layout). For rMATS MXE files, HUGO gene name (column 3), chromosome (column 4), strand (+/–, column 5), 0 based coordinates in columns 6–13, FDR (column 22), and inclusion level difference (column 25). In each case, all other columns are ignored. SpliceTools also requires line 1 to be a header, followed by individual lines with event data. Output from other programs can be hacked by transferring the appropriate elements to the appropriate columns, leaving other columns blank and including a header line. SpliceCompare requires the AS files to conform to the following suffixes: ‘_A3SS.MATS.JCEC.txt’, ‘_A5SS.MATS.JCEC.txt’, ‘_MXE.MATS.JCEC.txt’, ‘_RI.MATS.JCEC.txt’ and ‘_SE.MATS.JCEC.txt’. Text preceding the underscore should specify a unique name for the respective experiment.

### Gene set enrichment analysis (GSEA)

GSEA was performed using GSEA 3.0 (18) with the -permute gene set flag and the E2F1_Q6_01 and MYC_Q2 signatures.

### Resources

The SpliceTools suite is housed at https://github.com/flemingtonlab/SpliceTools. All rMATS AS output data for RBP, UPF1, SMG6, and SMG7 knockdown and indisulam, ms023, and CC115 treatment experiments can be downloaded at https://github.com/flemingtonlab/SpliceTools/tree/main/data.

## RESULTS

**Figure 1.**
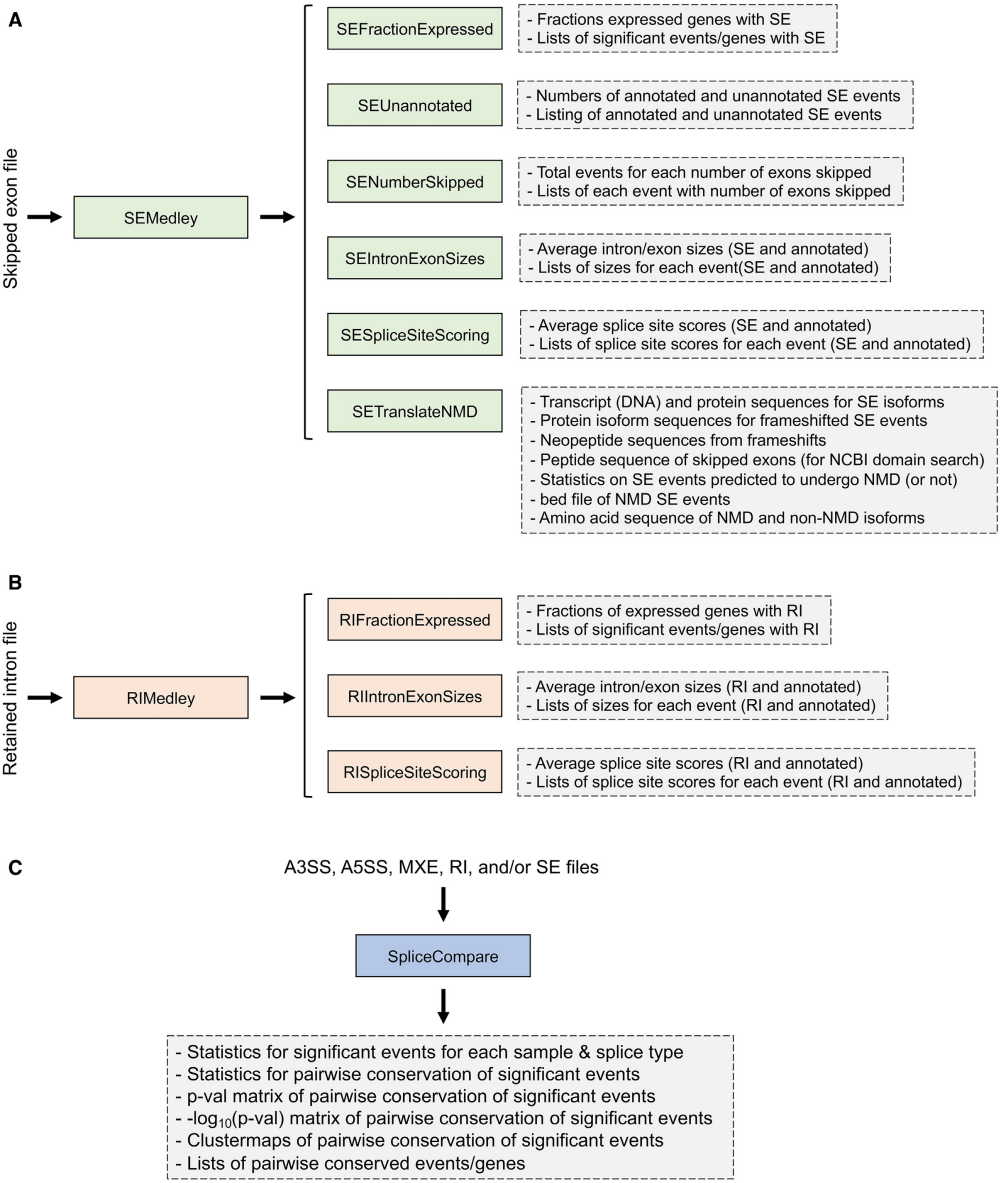
*SpliceTools schematic*. (**A**) The six core skipped exon analysis tools are shown with their respective outputs indicated to the right. Tools can either be used individually or all six can be called using one command with SEMedley. (**B**) Listing of core tools for retained intron analysis and their outputs are indicated. RIMedley can be used to run all three retained intron tools using a single command. (**C**) SpliceCompare analyzes any combination of A5SS, A3SS, MXE, RI, or SE files for comparative analyses of AS profiles across datasets. A parallel processing version of SpliceCompare is provided that utilizes 5 processors to simultaneously analyze A5SS, A3SS, MXE, RI and SE files if the user has installed the Parallel::Forkmanager module.

Alternative splicing is classified into five distinct types, A5SS (alternative 5’ splice site), A3SS (alternative 3’ splice site), MXE (mutually exclusive exons), RI (retained intron) and SE (skipped exon). While all five AS types play roles in normal cell signaling, RI and SE are frequently the most consequential under normal, pathologic and pharmacologic conditions, with exon skipping typically being the most common altered event type. Outside of using all five AS types to assess functional relationships between datasets (‘SpliceCompare’ (see below)), SpliceTools therefore focuses primarily on the investigation of RI and SE AS events (Figure 1).

### Datasets

We illustrate the utility of SpliceTools using four existing datasets. Knockdown of 186 known RNA binding proteins (RBPs), including splicing factors, in the HepG2 cell line from the ENCODE project (15) is used as a reference for assessing general characteristics, deciphering similarities, and determining mechanisms of AS. Disruption of nonsense mediated RNA decay (NMD) causes apparent AS changes through stabilization of alternatively spliced isoforms that normally undergo degradation through NMD. We used NMD inhibition data sets (inhibition by CC115) and knockdown of the NMD factors, UPF1, SMG6 and SMG7 (19) as a baseline for splicing inhibition through RBP knockdowns and pharmacologic splicing inhibition and to assess NMD prediction using the SETranslateNMD module. Pharmacologic splicing inhibition (8) using the RBM39 degrading sulfonamide, indisulam (20,21) and inhibition of type I protein arginine methyltransferases (ms023) (9,22) to inhibit arginine methylation of splicing factors is instructive for demonstrating the footprint of pharmacological inhibition on the transcriptome, for providing mechanistic predictions, for predicting impacted pathways, and to identify candidate neo-antigens with relevance to cancer immune therapy.

### Footprint of AS on cell transcriptome (SEFractionExpressed and RIFractionExpressed)

In physiologic cell signaling, cell fate can be determined by as little as a single AS event (1) but typically involves splicing alterations across groups of tens to thousands of genes (e.g. (23)). For investigational studies, assessing the AS transcriptome footprint addresses the overall impact of AS arising from genetic alterations or experimental or pharmacological manipulation. SEFractionExpressed and RIFractionExpressed determine the fraction of all expressed genes (expressed at minimum input TPM values) with significant changed SE or RI AS events between two conditions.

**Figure 2.**
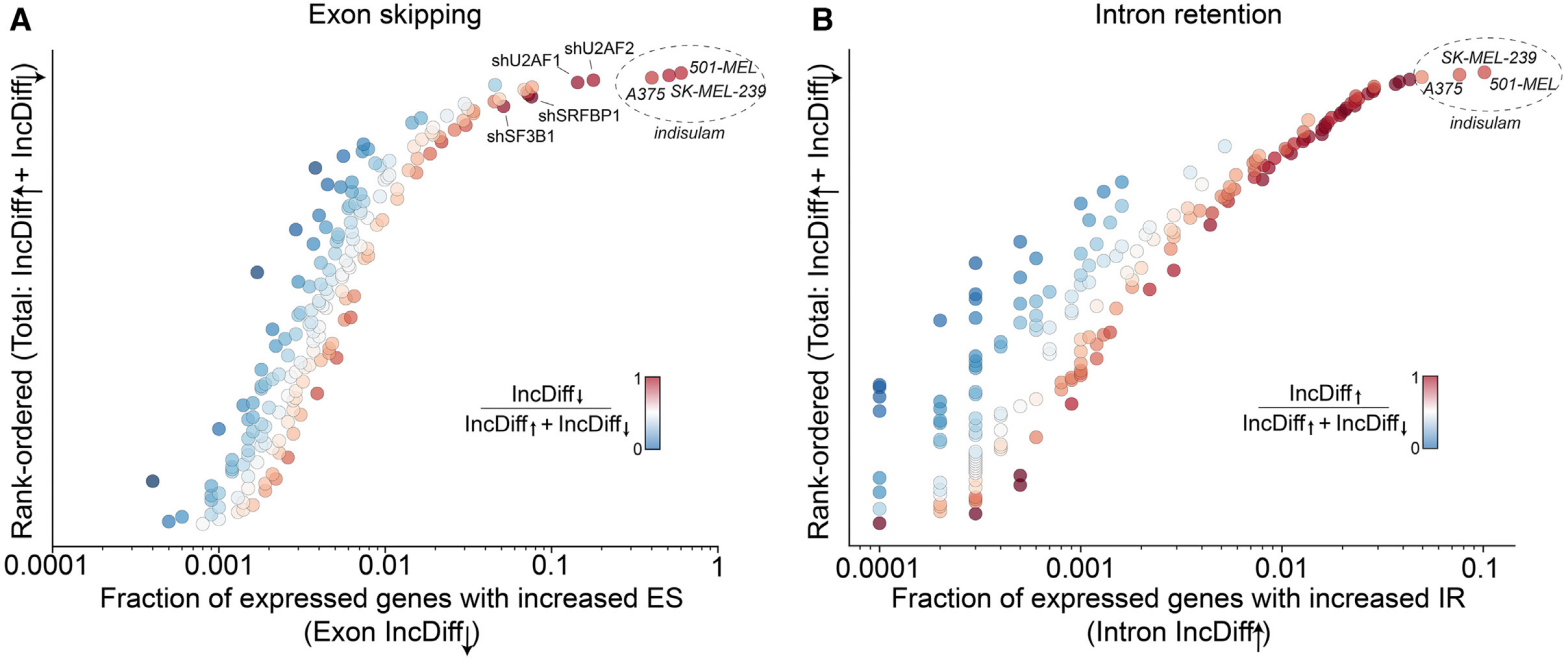
*Transcriptome footprint of SE and RI for RBP knockdowns and pharmacologic splicing inhibitors*. SEFractionExpressed and RIFractionExpressed were used with input parameters of minimum TPMs = 3 and an AS threshold FDR < 0.0005. Colors and intensities indicate the proportion of significantly changed AS events with increased exon skipping or intron retention. (**A**) fraction of expressed genes with increased exon skipping (–IncDiff (Inclusion Difference)). (**B**) fraction of expressed genes with increased intron retention (+IncDiff) events.

Comparing the induced SE footprint of the pharmacologic splicing inhibitors indisulam and ms023 (8) and the RBP knockdowns (15) illustrates the profound impact of indisulam treatment in all three cell lines tested (Figure 2). Treatment with indisulam caused statistically significant (FDR < 0.0005) increases in exon skipping in 40–60% of all genes expressed at a minimum of 3 TPMs in the control condition (Figure 2A). Among the RBP knockdowns, the two key U2 splice acceptor recognition factors, U2AF1 and U2AF2 have the largest footprint on the transcriptome (Figure 2A). Also notable among the RBP knockdowns, knockdown of the transcription factor, SRFBP1, which has not been previously linked to splicing, shows a significant level of increased exon skipping (Figure 2A). Findings such as this can prompt an investigator to validate novel findings and investigate further if warranted.

The transcriptome-wide impact of increased intron retention is considerably smaller than for exon skipping but is nevertheless substantial in indisulam treated cells, with treatment of 501-MEL cells causing increased intron retention in as many as ∼10% of expressed genes (Figure 2B). Together, SEFractionExpressed and RIFractionExpressed provide a macroscopic view of SE and RI by illuminating the footprint of AS on the cell transcriptome.

### Fraction unannotated SE events

**Figure 3.**
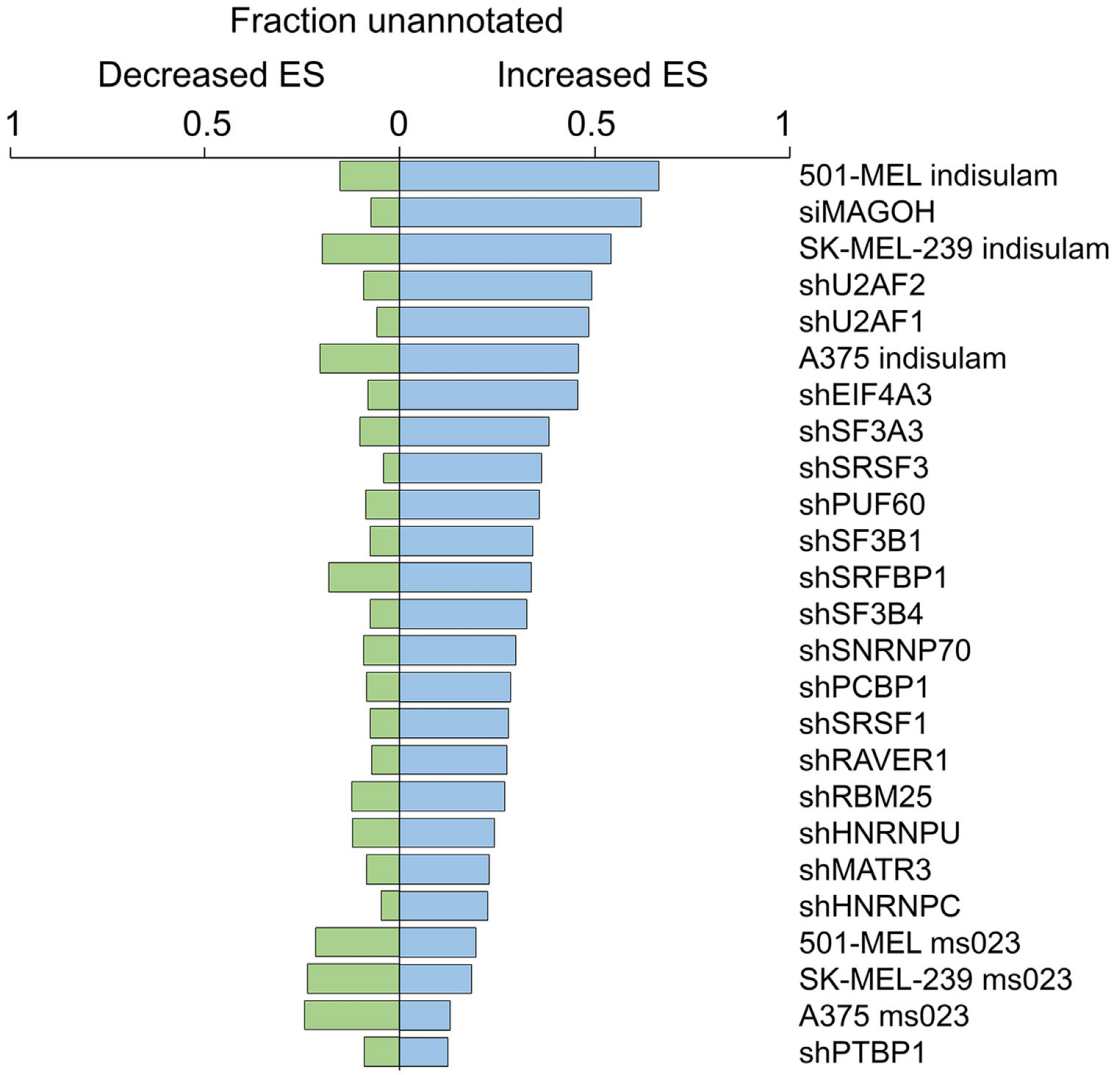
*FractionUnannotated*. Plots indicate the fraction of statistically significant (FDR < 0.0005) increased (blue) or decreased (green) exon skipping events that are not denoted in the input annotation file.

SEUnannotated determines the fraction of changed SE splice junctions that are not denoted in an input annotation file. This parameter can be an indicator guiding the discovery of novel splicing programs; such as our recent findings of a unique splicing program in blood naïve B-cells (24). This calculation can also provide supportive evidence for splicing disruption versus canonical splicing regulation. Under conditions where expression of core splicing factors such as MAGOH, U2AF1, and U2AF2 are inhibited or where the core U2 factor, RBM39 is degraded by treatment of cells with indisulam (25), between 45% and 66% of increased exon skipping events were not previously annotated (Figure 3). This contrasts with the smaller fraction of unannotated SE events occurring naturally under control conditions that are decreased under test conditions. This indicates that these splicing disruption conditions induce substantial novel exon skipping.

In contrast to indisulam which inhibits core splicing mechanisms, ms023 treatment inhibits the arginine methylation of splicing regulators such as HNRNPs. Accordingly, ms023 treatment and knockdown of the HNRNPU, HNRNPC and PTBP1 splicing regulators resulted in predominantly annotated increased skipping events (Figure 3), consistent with these treatments impacting splicing regulation rather than inhibiting core splicing functions.

In addition to outputting the fractions of annotated and unannotated events, SEUnannotated outputs listings of all unannotated splice junctions for cataloging novel splicing events. Together, SEUnannotated can identify novel splicing physiologies, it can provide supporting information for distinguishing splicing disruption from splicing regulation, and it annotates novel splicing events.

### Number skipped

**Figure 4.**
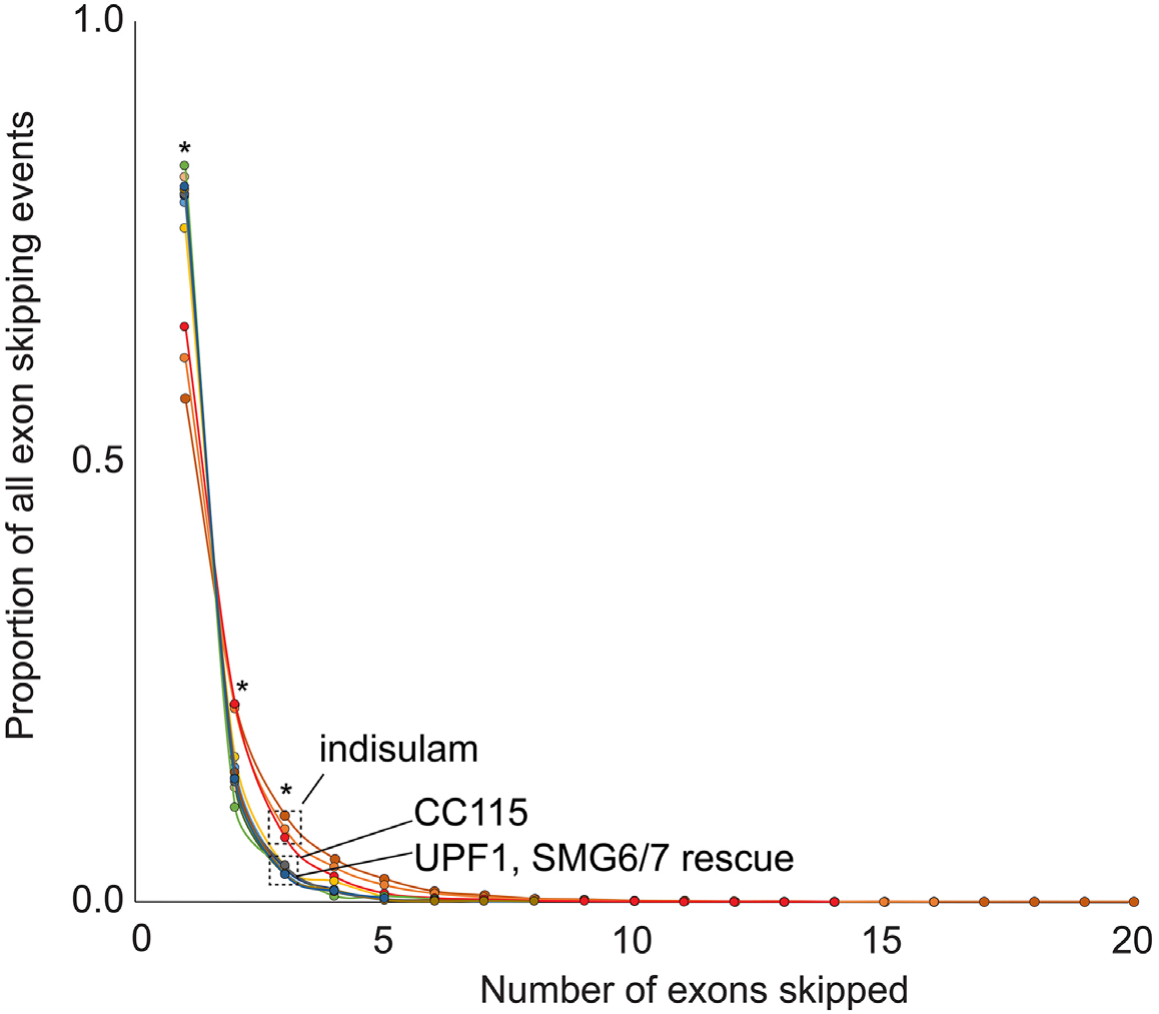
*NumberSkipped*. Distribution of multiple exon skipping across datasets. SENumberSkipped was run using an FDR cutoff of 0.0005. The plot includes experiments with >100 detected -IncDiff events (increase in skipping). The splicing inhibitor, indisulam, shows higher numbers of exons skipped. Increases in skipped isoform levels upon NMD inhibition (e.g. CC115 and SMG6/7 knockdown), more frequently involve single exon skipping.

Experimental and pharmacologic splicing disruption leads to aberrant exon skipping. Depending on the degree of disruption, this can lead to an increased likelihood of skipping multiple exons. In contrast, physiologic regulation of exon skipping is more likely to lead to single or few numbers of exons skipped. SENumberSkipped tabulates the number of annotated intervening exons harbored between the boundaries of the skip junctions. As shown in Figure 4, indisulam treatment resulted in a higher proportion of skipping events with multiple skipped exons compared to those observed with SE isoforms that increase in abundance when NMD factors are knocked down or when NMD is inhibited through treatment with CC115. This highlights the disruptive nature of splicing inhibition compared to physiological regulation of SE isoform abundance through NMD.

### Intron and exon sizes

Splicing of nascent transcripts is dependent on the recruitment of U1 factors to upstream splice donor sequences, U2 factors to downstream splice acceptor sequences, splicing enhancers to primarily exons, and splicing suppressors to primarily introns. The recruitment of these factors generally occurs co-transcriptionally and the timely assembly of upstream U1 donor and downstream U2 acceptor complexes favors the splicing of consecutive exons over exon skipping. Suboptimal splice site or enhancer binding sequences can lead to slower loading of splicing factors and transcription through downstream exon acceptor sequences before the upstream consecutive exons are spliced. This results in the competitive availability of downstream splice acceptor sequences for splicing to the upstream splice donor and an increased likelihood of exon skipping. Another factor impacting the timely loading of U1 and U2 factors is the duration of RNA Pol II transit across exon/intron sequences. Intron length is one of the key structural features influencing RNA POL II transit time, with shorter introns facilitating rapid transit across exon/intron cassettes and increased competitive availability of downstream splice acceptor sequences.

Exon sizes are mechanistically linked to splicing through differential dependence on binding splicing enhancers. Restricted by the limitation of maintaining amino acid coding sequences, the evolution of exon sequences towards binding splicing enhancers is restrained. Because of this restriction in evolutionary adaptation, the greater amount of genetic material intrinsic to long exons increases the probability of adopting splicing enhancer binding sequences. Since splice acceptors are specified by the combined influence of both intronic U2 binding sequences and exonic splicing enhancer binding, smaller exons often rely more heavily on high quality U2 factor binding sequences whereas longer exons are frequently more dependent on splicing enhancer binding. Because of the relationships between intron/exon sizes and mechanisms driving splicing, assessing intron and exon sizes across experimental conditions is pertinent to the molecular basis of associated AS.

SEIntronExonSizes determines the sizes of the upstream exon, upstream intron, skipped exon, downstream intron and the downstream exon for all statistically significant SE events for both increased and decreased exon skipping. As a baseline, it also queries an input annotation file to generate the same information for potential skipping events across an annotated transcriptome. A summary file with the average intron and exon sizes is generated as well as listings of sizes for each individual event (for individual event level analyses, for alternative statistical treatments, and for plotting size distributions).

**Figure 5.**
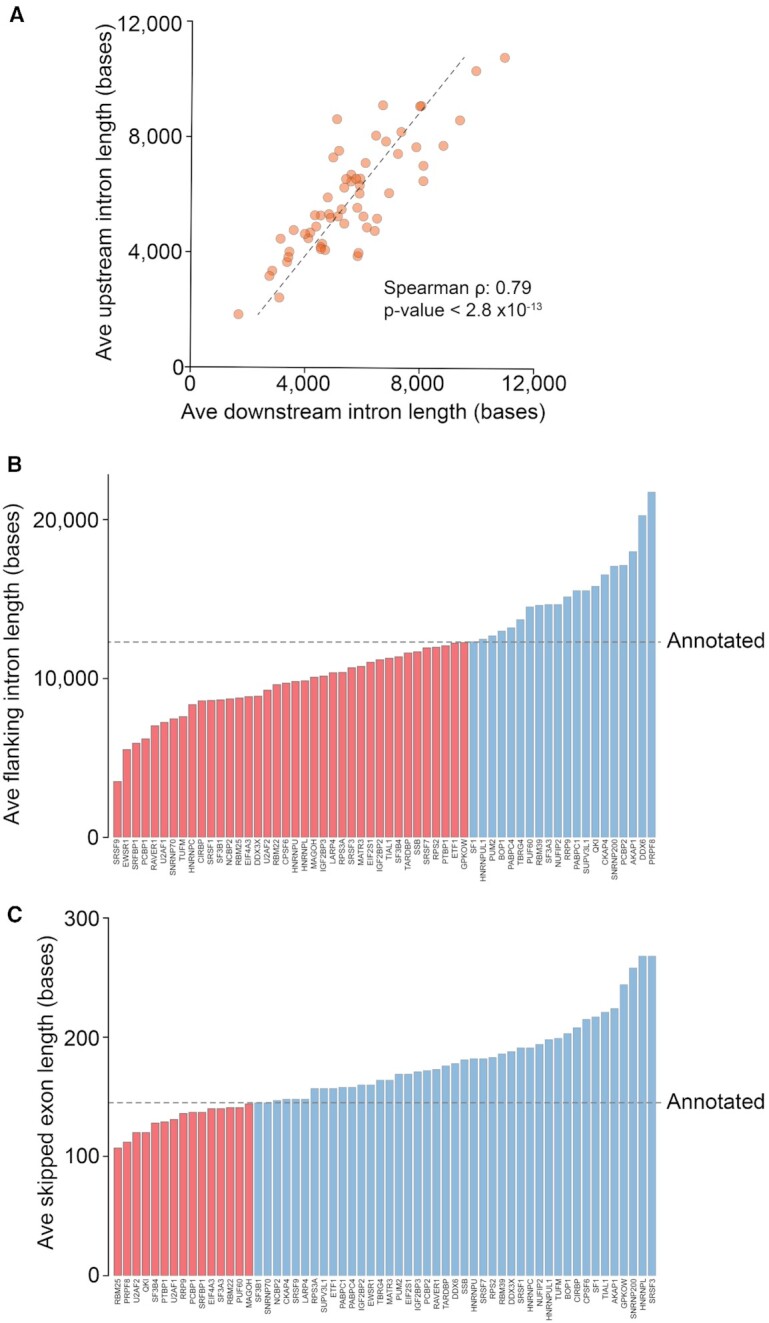
*IntronExonSizes*. Distributions of flanking intron and skipped exon sizes across ENCODE RBP knockdown dataset. SEIntronExonSizes was run using an FDR cutoff of 0.0005. Average sizes for experiments with greater than 100 -IncDiff (increase in skipping) events were plotted. (**A**) Correlation between upstream and downstream flanking intron sizes. Average flanking intron (**B**) and skipped exon (**C**) sizes for events with increased skipping are plotted for each knockdown. Average sizes for potential skipping events, derived from input annotation file, are indicated by the dashed ‘Annotated’ line.

Using SEIntronExonSizes to analyze ENCODE RBP knockdowns that cause at least 100 increased exon skipping events, a strong correlation between the upstream and downstream intron sizes is evident across the different RBP knockdowns (Figure 5A). This suggests a functional relationship between upstream and downstream intron sizes and it provides evidence for a mechanistic linkage between flanking intron sizes and the unique mechanisms driving skipping by individual RBP knockdowns. Despite a wide range of average intron sizes across the knockdowns, the majority of knockdowns showed increased exon skipping with shorter average flanking intron sizes, including knockdown of the core splicing effectors, U2AF1, U2AF2, SF3B1 and the splicing enhancers, SRSF1, SRSF3, SRSF7 and SRSF9 (Figure 5B). This is consistent with the rapid transit of RNA POL II across short introns limiting the time for depleted splicing factors to bind upstream donor and skipped exon acceptor sequences, leading to the competitive availability of the downstream acceptor and exon skipping.

In contrast to intron sizes, increased exon skipping due to RBP knockdowns occurs more frequently at larger skipped exons. Consistent with splicing to larger exons being more reliant on splicing enhancers, knockdown of each of the four splicing enhancers, SRSF1, SRSF3, SRSF7, and SRSF9 causes skipping at exons that are larger than average (Figure 5C). On the other hand, increased exon skipping caused by knockdown of most of the core U2 factors, including U2AF1, U2AF2, SF3A3, SF3B4, EIF4A3, PUF60 and MAHOH occurs at smaller skipped exons (Figure 5C). This is consistent with smaller exons having a greater reliance on high quality flanking U2 factor intronic binding sequences where knockdown of U2 factors have a greater impact on skipping. Together, these results highlight some of the mechanistic principles underlying the relationship between intron/exon sizes and the distinct functions of RBPs. More importantly, they illustrate the utility of comparative analyses using SEIntronExonSizes to implicate classes of splicing factors (e.g. splicing enhancers, U2 factors, etc.) in splicing changes induced by an investigator's test conditions.

### Splice site scoring

The binding of U1 splice donor factors and U2 splice acceptor factors to primary transcripts depend on the quality of the splice junction sequences (as well as the upstream polypyrimidine tract for the splice acceptor). SESpliceSiteScoring and RISpliceSiteScoring use the MaxEntropy algorithm developed by the Burge lab (26) to globally assess the quality of all splice junction sequences for significantly increased and decreased SE and RI events. For reference, an input annotation file is used to generate splice site scores for potential SE and RI events across an annotated transcriptome.

**Figure 6.**
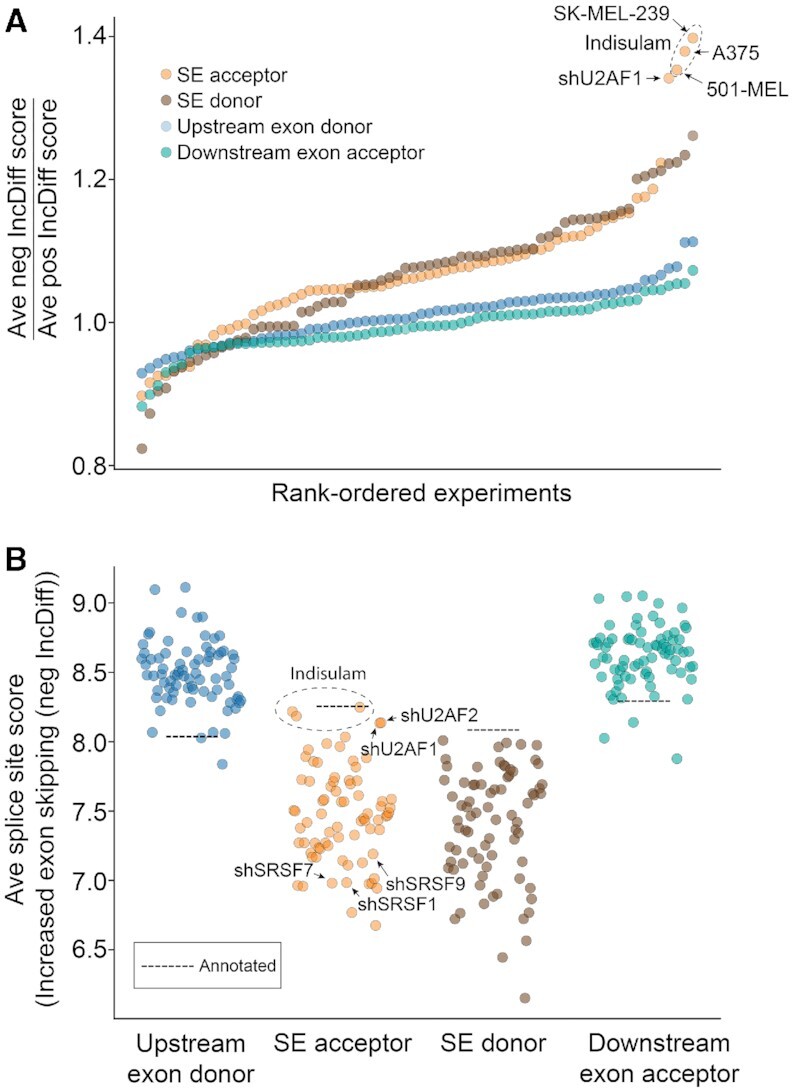
*SpliceSiteScoring*. Average splice site scores for upstream exon donor, skipped exon acceptor, skipped exon donor and downstream exon acceptor were determined for events with increased exon skipping (FDR < 0.0005) in RBP knockdowns and indisulam or ms023 treated cells. Only experiments with greater than 100 significant events were considered. A) Ratio of splice site scores for significant increased exon skipping events (–IncDiff) to significant decreased exon skipping events (+IncDiff). B) Average splice site scores for increased skipping events. Dashed lines provide reference scores for potential skipping configurations derived from an input annotation file.

Using SESpliceSiteScoring, we assessed splice site scores for the ENCODE RBP knockdown and indisulam treatment datasets. This analysis revealed average splice site scores for flanking upstream splice donors and downstream splice acceptors that were higher than annotated SE configurations for both increased (Figure 6B) and decreased (Supplementary Figure S1) SE events. In contrast, the average splice site scores for the skipped exon splice acceptor and donor sites are lower than average annotated SE configurations (Figure 6B and Supplementary Figure S1). Both strong upstream donor/downstream acceptor and weak skipped exon acceptor/donor sites are consistent with a propensity for exon skipping, whether it occurs naturally (e.g. for decreased exon skipping events) or whether it is induced (increased exon skipping) by splicing inhibition.

To decipher mechanistic differences across splicing disruption conditions, we first investigated the ratio of splice site scores for increased versus decreased exon skipping. The ratios for flanking upstream splice donor and downstream splice acceptor sites varied marginally across different splicing inhibition conditions (Figure 6A). This suggests that while high flanking exon splice site scores tend to promote exon skipping, there is little discernable mechanistic difference in the roles of these sites between induced and decreased exon skipping. In contrast, a substantially greater variance was observed for the skipped exon splice acceptors and donors across the different RBP knockdowns and pharmacologic splicing inhibition conditions (Figure 6A). Further, we noted a statistically significant correlation between the skipped exon acceptor and donor scores across conditions (Supplementary Figure S2). These findings support a relationship between the mechanistic basis of different RBPs (and pharmacologic splicing inhibitors) and the quality of the skipped exon splice acceptor and donor.

We next investigated the relationship between the skipped exon acceptor scores and splicing inhibition conditions in more detail. Consistent with larger skipped exons being more dependent on splicing enhancer binding for acceptor definition (Figure 5C), the average skipped exon splice acceptor scores are low for the splicing enhancer SRSF1, SRSF7, and SRSF9 knockdowns (Figure 6B). In contrast, knockdown of U2AF1 and U2AF2, which bind to splice acceptor sequences, have less dependence on splicing enhancers (Figure 5C) and a greater reliance on high quality splice acceptor sites (Figure 6B). RBM39 inhibition through indisulam treatment similarly caused skipping of exons with higher splice site scores (Figure 6B), consistent with the involvement of RBM39 in the U2 complex. In conjunction with intron/exon sizes, the comparative assessment of splice site scores links experimentally induced splicing changes to the unique properties of different classes of splicing factors, providing insights into the underlying mechanisms driving altered splicing.

### SpliceCompare

SpliceCompare determines similarities in alternative splicing profiles across experiments for all five AS types to investigate mechanistic relationships and the molecular basis of splicing alterations elicited by a user's experimental condition. It also provides a convenient summary file with numbers of positive, negative and total significant A5SS, A3SS, MXE, RI and SE events for each experimental condition (Supplementary File S2). Using summary output from SpliceCompare, Supplementary Figure S3 illustrates the highly disruptive nature of indisulam where we observed extensive AS across all 5 types and 15000 to 35000 increased exon skipping events (Supplementary Figure S3).

The core comparison function of SpliceCompare determines all pair-wise overlaps for significant positive and negative inclusion difference (IncDiff) events across conditions, outputting a summary file listing the total number of significant events for each condition and the number of common events for increased, decreased and total (increased plus decreased) IncDiff events. It then performs a hypergeometric test to approximate the significance of overlap using total events detected across both conditions as a surrogate for population size. Matrix files with overlap p-values are generated to facilitate detailed analyses and further statistical treatments, such as multiple testing adjustment. Corresponding -log_10_(p-value) matrix files are also generated for input into a user's preferred clustering algorithm. Lastly, default clustering of -log_10_(p-values) is performed to provide an initial graphical illustration of results.

**Figure 7.**
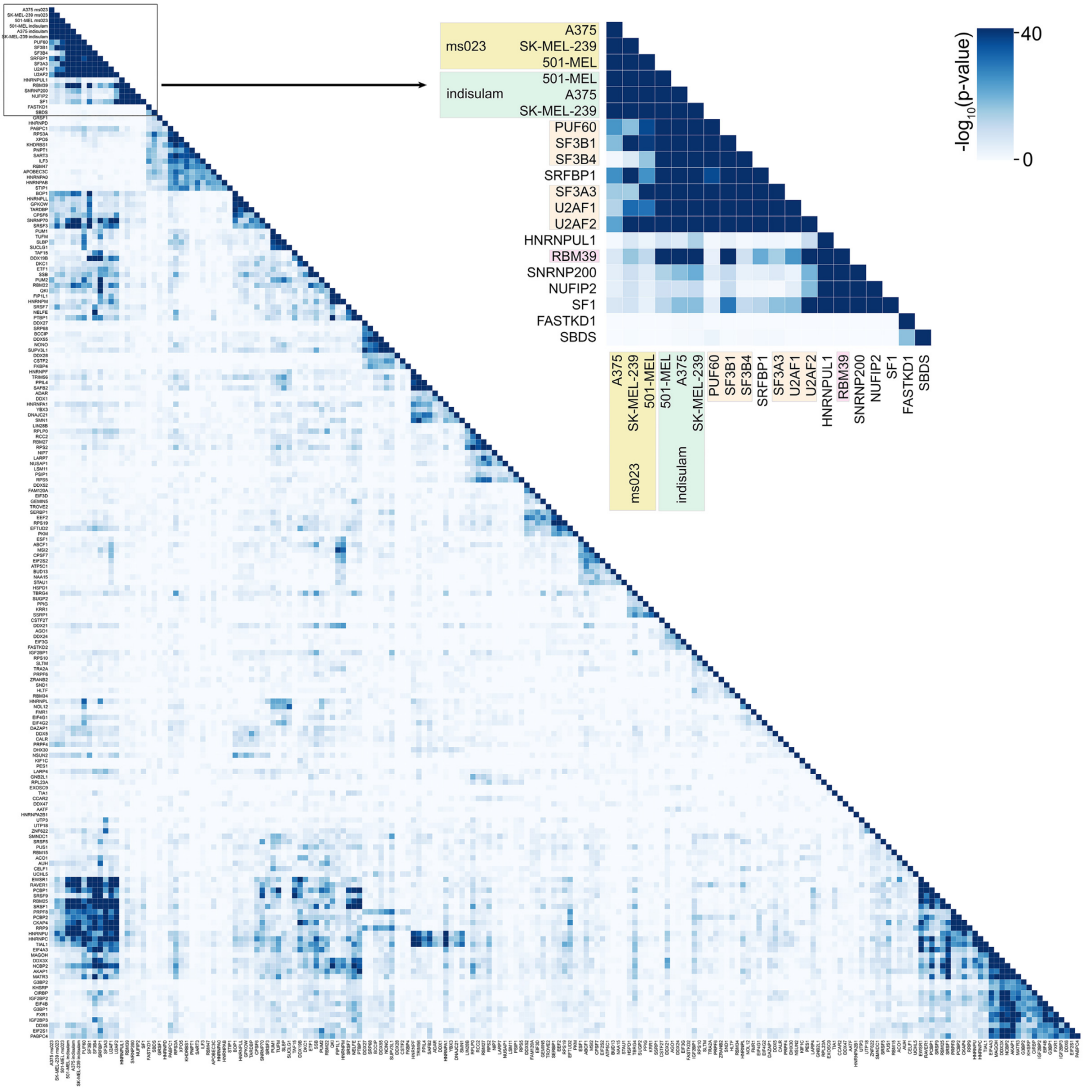
*SpliceCompare*. Comparative analysis of common increased exon skipping events (FDR < 0.0005) for RBP knockdowns and pharmacologic splicing inhibition.

As an example, Figure 7 displays results from an analysis of common increased exon skipping events across the RBP knockdowns and the pharmacological inhibitors, indisulam and ms023. The cutout in Figure 7 shows the high degree of overlap for knockdown of the U2 assembly factors, U2AF1, U2AF2, SF3B1, SF3B3, SF3B4 and PUF60. Notably, indisulam treatment, which causes the degradation of the U2 associated factor, RBM39 shows strong overlap with knockdown of U2 assembly factors whereas ms023, which inhibits the arginine methylation of splicing suppressors, shows less overlap with U2 assembly factors (Figure 7). Included in the ENCODE RBP knockdown group is RBM39. Nevertheless, RBM39 knockdown in HepG2 cells resulted in >40-fold fewer increased exon skipping events than indisulam treatment in any of the three cell lines tested. This may be the result of more impactful decreases in RBM39 levels by indisulam mediated protein degradation than by RBM39 shRNA knockdown (which is 31% of control at the RNA level) or it may be due to additional mechanisms through which indisulam alters splicing. Despite this difference in overall impact on exon skipping, we observed a high similarity between RBM39 knockdown and indisulam treatment but low similarity between RBM39 knockdown and ms023 (Figure 7). Together, these results illustrate how SpliceCompare can be used to investigate the mechanistic underpinnings of splicing alterations caused by a user's experimental conditions.

### SETranslateNMD

**Figure 8.**
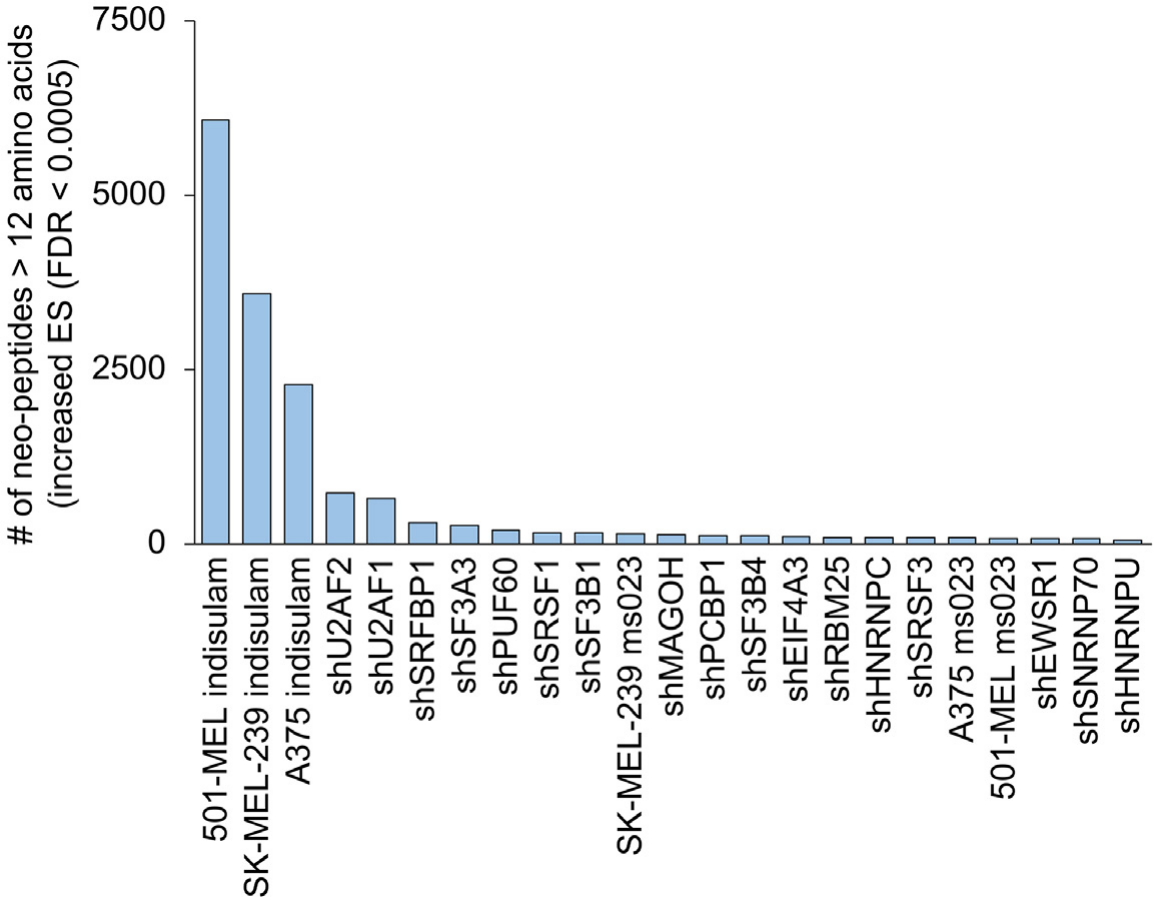
*Neopeptide prediction from SETranslateNMD*. Numbers of neopeptides longer than 12 amino acids long predicted from frameshifts generated by increased exon skipping (FDR < 0.0005) across the top 23 RBP knockdowns and pharmacologic splicing inhibition.

SETranslateNMD provides output for interrogating the functional impact of exon skipping caused by a user's test condition. Predicted protein isoform sequences are provided in fasta format for all significant exon skipping events. To investigate the potential consequences of treatment conditions on the adaptive immune response, listings of all potential neopeptide sequences caused by frameshifts are also generated. Comparing the possible immunological impact of pharmacological splicing disruption used in the Lu et al (8) study and the RBP knockdowns from the ENCODE study (15), the substantial potential of indisulam to elicit an adaptive immune response is apparent with between 2294 and 6082 neopeptides >12 amino acids long predicted to arise from frameshifted increased exon skipping events (Figure 8).

To predict the functional consequences of exon skipping alterations, SETranslateNMD identifies all transcripts with exon skipping changes that are predicted to undergo NMD and thereby decrease expression of the respective gene. To help the user interpret the functional impact of in-frame exon skipping events, SETranslateNMD also outputs the amino acid sequence of all in-frame skipped exons in a format suitable for querying the NCBI conserved protein domain search database (https://www.ncbi.nlm.nih.gov/Structure/bwrpsb/bwrpsb.cgi) (27).

**Figure 9.**
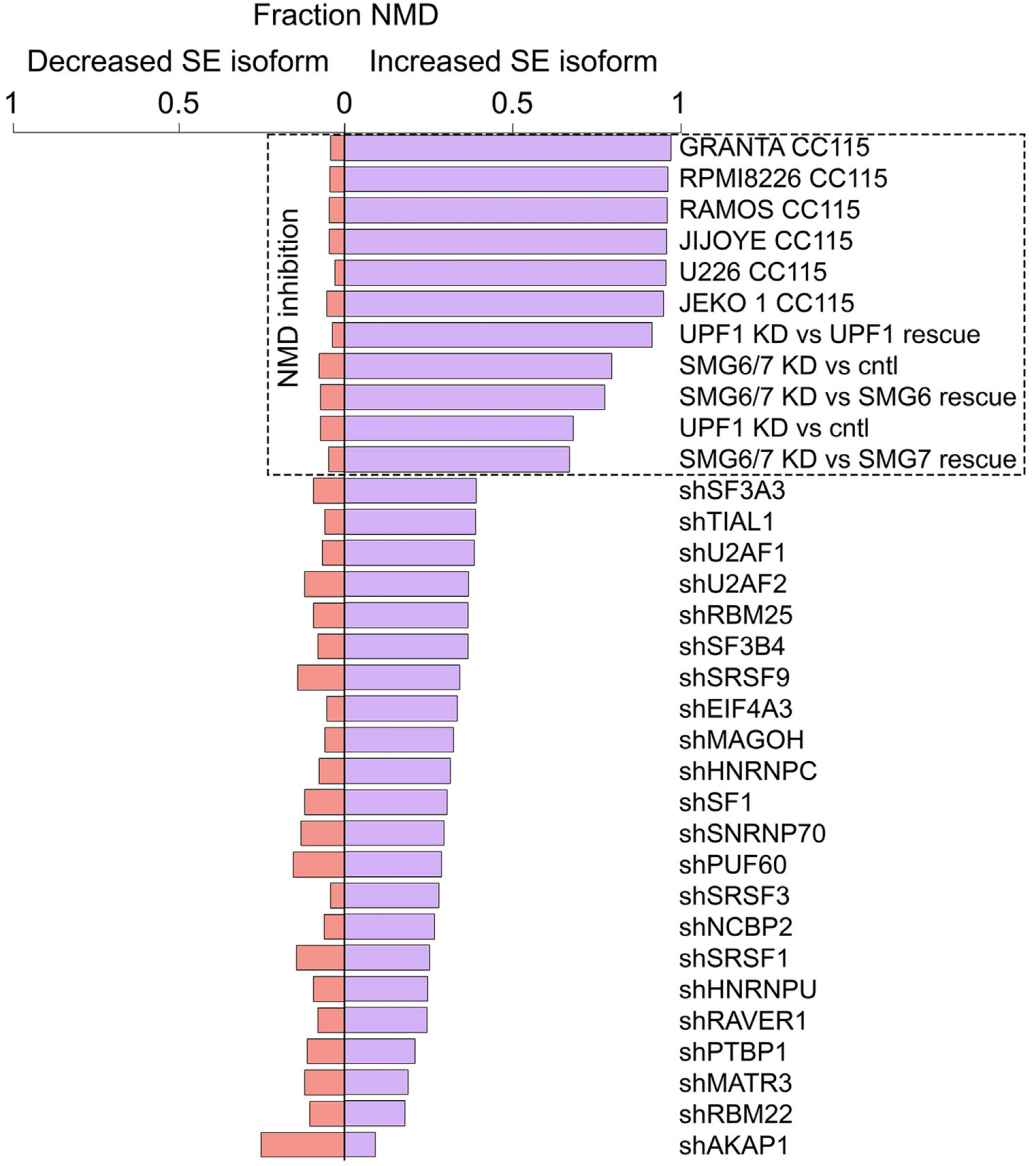
*NMD prediction from SETranslateNMD*. Fraction predicted NMD isoforms are plotted for NMD inhibition and RBP knockdown experiments with a minimum of 100 increased and 100 decreased (FDR < 0.0005) skipped exon isoform level changes. SE = skipped exon.

For NMD prediction, frameshifted SE transcripts with a premature translation termination codon located >50 bases upstream from an exon-exon junction are considered NMD candidates. Using NMD summary statistics from SETranslateNMD analysis of RBP knockdowns and NMD inhibitors, relatively few baseline exon skipping events with reduced exon skipping under test conditions are predicted to undergo NMD (Figure 9, ‘decreased SE isoform’). In contrast, exon skipping events that are induced by the knockdown of splicing regulators display greater numbers of skipped exon isoforms that are predicted to undergo NMD (Figure 9). Most notably, 67–97% of SE isoforms showing increased abundance when NMD is inhibited are predicted to undergo NMD (Figure 9), illustrating the ability of SETranslateNMD to detect NMD targeted transcripts. Strikingly, treatment of cells with CC115, which is most commonly used as an mTOR/DNA-PK inhibitor (28,29), results in 94–97% of increased SE isoforms predicted to be NMD targets (Figure 9), higher than that observed with the knockdown of NMD factors (Figure 9). This illustrates the remarkable specificity of CC115 for altering the levels of NMD transcript isoforms.

**Figure 10.**
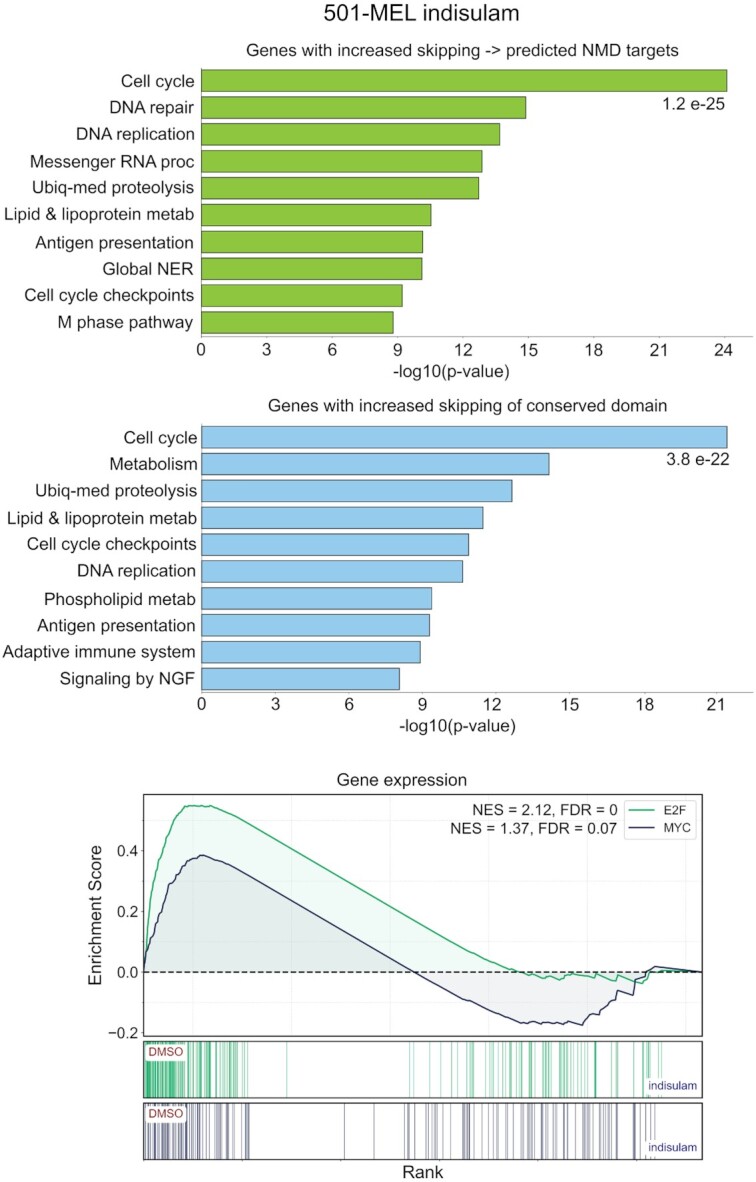
Predicting the functional impact of exon skipping. Pathway analysis of genes with increased exon skipping predicted to undergo NMD (upper panel) and with skipped conserved domain sequences (middle panel) were analyzed by Enrichr (https://maayanlab.cloud/Enrichr/) with the BioPlanet 2019 pathway database. Gene set enrichment analysis (GSEA) of gene expression (lower panel) was performed using E2F and MYC target signatures.

In addition to summary statistics, SETranslateNMD outputs a bed file with the coordinates of all exon skipping events predicted to undergo NMD and files listing all genes with at least one changed isoform predicted to undergo NMD. Using the NMD gene list for increased exon skipping in cells treated with indisulam, pathway enrichment analysis (Enrichr (30–32)) showed statistically significant enrichment of cell cycle genes (Figure 10 and Supplementary Figures S4 and S5). We then used the amino acid sequences of skipped exons from in-frame increased skipping events caused by indisulam and submitted them to NCBI batch conserved domain search. Pathway enrichment analysis of all genes with skipped exons containing a conserved domain similarly showed enrichment of cell cycle genes (Figure 10 and Supplementary Figures S4 and S5). These analyses predict that the substantial level of increased exon skipping induced by indisulam influences the cell cycle. To test whether the cell cycle is, in fact, altered by indisulam treatment, we performed Gene Set Enrichment Analysis (GSEA) (18) to assess changes in the expression of the cell cycle transcription factors, E2F and MYC targets. These analyses showed decreased E2F and MYC activity in cells treated with indisulam, demonstrating a functional impact of indisulam on the cell cycle (Figure 10 and Supplementary Figures S4 and S5). Notably, these results are consistent with the diminished cell growth detected at days 5–7 of indisulam treatment by Lu et al (27).

Overall, SETranslateNMD provides multiple outputs pertaining to mRNA translation that are useful for interpreting the significance of SE changes. By assessing the fraction of increased exon skipping events predicted to undergo NMD, SETranslateNMD can be used to infer whether a test condition influences the NMD pathway. SETranslateNMD identifies candidate neo-peptides with potential significance to adaptive immune responses. SETranslateNMD identifies transcripts that are predicted to be degraded by NMD and transcripts with in-frame exon skipping events that splice-out potential important functional domains for assessing the potential functional impact of both the out-of-frame and in-frame sets of SE events on cell regulatory pathways. Lastly, lists of each NMD candidates and each in-frame skipped exon conserved domain excision can be used to investigate the functional significance of exon skipping at the individual event level. Together, SETranslateNMD provides broad and fertile data to facilitate the development of hypotheses regarding the functional influence of alternative splicing on cell signaling and cell fate.

## DISCUSSION

We previously developed a tool (SpliceV (33)) to generate publication quality visualization of gene level splicing alterations. Here, we report SpliceTools, a suite of tools designed to help investigators studying alternative splicing overcome the bottleneck between acquisition of differential splicing data and interpretation. The SpliceTools suite provides summary statistics and general characteristics of alternative splicing, it provides structural features and comparative approaches to investigate the mechanistic bases of alternative splicing, and it provides output that can be interrogated for predicting the biological impact of alternative splicing. Each of the ‘SE’ and ‘RI’ tools can be executed individually or all respective tools can be executed through a single command using SEMedley or RIMedley (Figure 1). Also included in the SpliceTools suite are ‘batch’ submission scripts for each of the SE or RI tools that facilitate the analysis of multiple differential splicing conditions with a single command.

SpliceCompare performs comparative analyses across experimental conditions to assess AS similarities for one or more of the five alternative splice types. A parallel processing version of SpliceCompare is also included (SpliceCompareParallel (requires that the perl module, Parallel::ForkManager)) that directs each splice type analysis to a distinct processor. SpliceCompare also includes the option to output lists of common increased, decreased, and total inclusion difference splicing alterations for each pairwise comparison (notably, this option results in 10s to 100s of thousands of output files when large numbers of conditions are analyzed, in which case the user may wish to suppress this output). This provides the user with information for downstream analyses investigating structural features unique to overlapping events.

Lastly, we also provide rMATS (10–12) AS output files for all datasets reported in this manuscript (https://github.com/flemingtonlab/SpliceTools/tree/main/data) as a resource to assist investigators in interrogating mechanisms of splicing alterations through comparative analyses. Together, the SpliceTools suite and this associated resource equips any investigator with basic command-line skills with a simple and robust framework to generate a wide array of summary statistics and to generate mechanistic and functional hypotheses.

## DATA AVAILABILITY

Data are available in the following repositories: https://github.com/flemingtonlab/SpliceTools/tree/main/data and https://doi.org/10.5281/zenodo.7603628.

## Supplementary Material

gkad111_Supplemental_Files
