# Supplementary material for: SpliceTools, a suite of downstream RNA splicing analysis tools to investigate mechanisms and impact of alternative splicing
Source: Nucleic Acids Res. 2023 Mar 2;51(7):e42. doi: 10.1093/nar/gkad111 (PMC10123099; doi:10.1093/nar/gkad111)
Supplement: gkad111_Supplemental_Files [file gkad111_supplemental_files.zip › Supplemental figs nar2022.pdf]

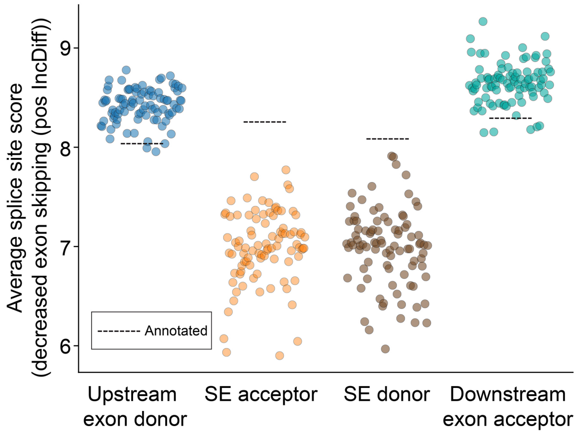

***Fig S1**. SESpliceSiteScoring - decreased SE events*. Average splice site scores for upstream exon donor, skipped exon acceptor, skipped exon donor and downstream exon acceptor were determined for decreased exon skipping events (FDR < 0.0005) for RBP knockdowns and indisulam or ms023 treated cells with greater than 100 significant events. Dashed lines provide reference scores for potential skipping configurations derived from an input annotation file.

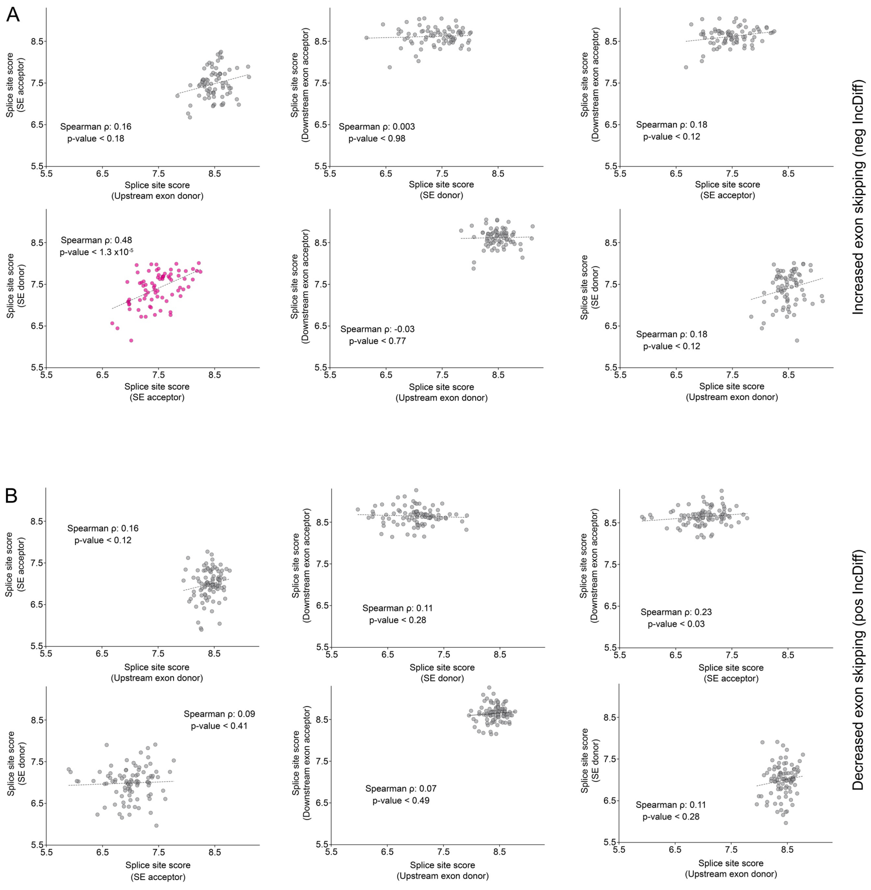

Fig S2*. Correlation analyses of average splice site scores*. Spearman correlation analyses of average splice site scores for upstream exon donor, skipped exon acceptor, skipped exon donor and downstream exon acceptor for statistically significant (FDR < 0.0005) increased exon skipping events for RBP knockdowns and indisulam or ms023 treated cells with greater than 100 significant events. Pink dots indicate a correlation p-value < 0.001.

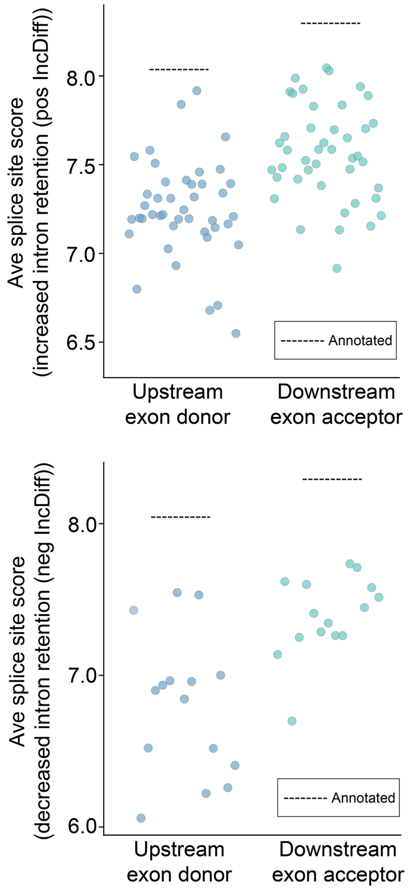

Fig S3*. Summary statistics of AS from SpliceCompare.* Data from statistically significant events (FDR < 0.0005) are plotted for all 5 AS types for pharmacologic splicing inhibitor datasets.

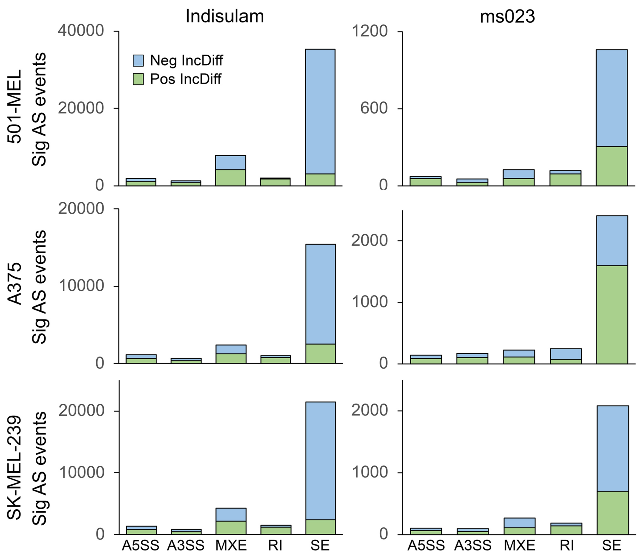

Fig S4. *Predicting the functional impact of exon skipping in SK-MEL-239 cells.* Pathway analysis of genes with increased exon skipping predicted to undergo NMD (upper panel) and with skipped conserved domain sequences (middle panel) were analyzed by Enrichr (https://maayanlab.cloud/Enrichr/) with the BioPlanet 2019 pathway database. Gene set enrichment analysis (GSEA) of gene expression (lower panel) was performed using E2F and MYC target signatures.

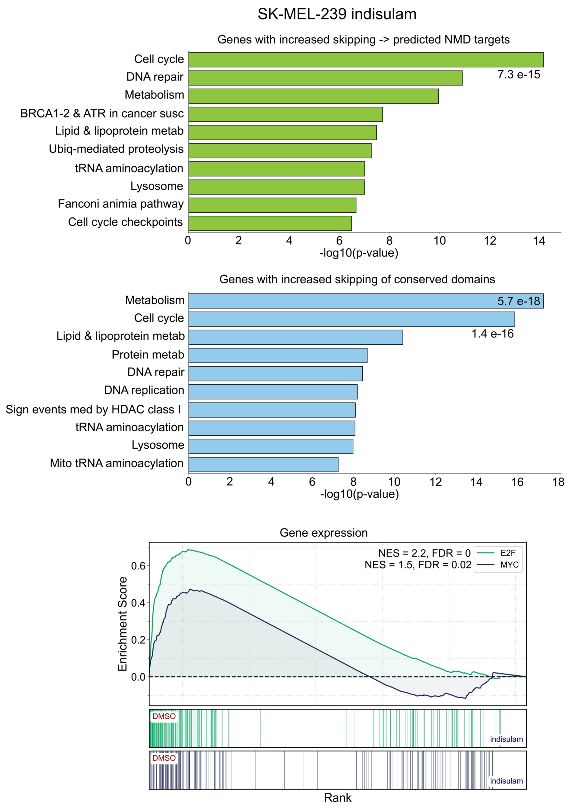

Fig S5. *Predicting the functional impact of exon skipping in A375 cells.* Pathway analysis of genes with increased exon skipping predicted to undergo NMD (upper panel) and with skipped conserved domain sequences (middle panel) were analyzed by Enrichr (https://maayanlab.cloud/Enrichr/) with the BioPlanet 2019 pathway database. Gene set enrichment analysis (GSEA) of gene expression (lower panel) was performed using E2F and MYC target signatures.
